# Correction: Optimal length and temporal resolution of dynamic contrast-enhanced MR imaging for the differentiation between prostate cancer and normal peripheral zone tissue

**DOI:** 10.1371/journal.pone.0322895

**Published:** 2025-04-22

**Authors:** Marius Hellstern, Carlos Martinez, Christopher Wallenhorst, Dirk Beyersdorff, Lutz Lüdemann, Marc-Oliver Grimm, Ulf Teichgräber, Tobias Franiel

The second affiliation for the first author is missing. Marius Hellstern is also affiliated with #6: Institut für Diagnostische und Interventionelle Radiologie, Universitätsklinikum Jena, Jena, Germany.

## References

[1] Hellstern M, Martinez C, Wallenhorst C, Beyersdorff D, Lüdemann L, Grimm M-O, et al. Optimal length and temporal resolution of dynamic contrast-enhanced MR imaging for the differentiation between prostate cancer and normal peripheral zone tissue. PLoS One. 2023;18(6): e0287651. doi: 10.1371/journal.pone.0287651 37352312 PMC10289347

