# Supplementary material for: Optimal length and temporal resolution of dynamic contrast-enhanced MR imaging for the differentiation between prostate cancer and normal peripheral zone tissue
Source: PLoS One. 2023 Jun 23;18(6):e0287651. doi: 10.1371/journal.pone.0287651 (PMC10289347; doi:10.1371/journal.pone.0287651)
Supplement: S1 File — (DOCX) [file pone.0287651.s001.docx]

**Supporting information**

**S1 Table. C-statistics from cross validation by measurement length and temporal resolution - Sensitivity analysis using covariates from best model.**

| **Measurement length [Minutes]** | **Temporal resolution [Seconds]** | | | | | | |
| --- | --- | --- | --- | --- | --- | --- | --- |
|  | **1.6** | **3.2** | **4.8** | **6.4** | **8.0** | **16.0** | **30.4** |
| 2.0 | 0.702 | 0.649 | 0.647 | 0.669 | 0.709 | 0.700 | 0.700 |
| 2.5 | 0.703 | 0.671 | 0.665 | 0.666 | 0.743 | 0.731 | 0.703 |
| 3.0 | 0.702 | 0.671 | 0.649 | 0.686 | 0.705 | 0.694 | 0.700 |
| 3.5 | 0.717 | 0.662 | 0.646 | 0.711 | 0.717 | 0.691 | 0.708 |
| 4.0 | 0.717 | 0.657 | 0.680 | 0.682 | 0.708 | 0.697 | 0.689 |
| 4.5 | 0.721 | 0.689 | 0.667 | 0.695 | 0.694 | 0.694 | 0.688 |
| 5.0 | 0.690 | 0.687 | 0.688 | 0.669 | 0.724 | 0.687 | 0.698 |
| 5.5 | 0.701 | 0.683 | 0.659 | 0.705 | 0.700 | 0.704 | 0.704 |
| 6.0 | 0.694 | 0.651 | 0.649 | 0.667 | 0.705 | 0.694 | 0.714 |
| 6.5 | 0.693 | 0.689 | 0.694 | 0.714 | 0.731 | 0.708 | 0.703 |
| 7.0 | 0.742 | 0.672 | 0.658 | 0.665 | 0.715 | 0.684 | 0.705 |
| 7.5 | 0.722 | 0.656 | 0.674 | 0.700 | 0.711 | 0.700 | 0.689 |
| 8.0 | 0.699 | 0.674 | 0.683 | 0.663 | 0.724 | 0.708 | 0.720 |
| 10.0 | 0.717 | 0.684 | 0.682 | 0.675 | 0.705 | 0.704 | 0.692 |
| 12.0 | 0.737 | 0.654 | 0.676 | 0.666 | 0.707 | 0.703 | 0.683 |
| 13.0 | 0.732 | 0.692 | 0.691 | 0.666 | 0.723 | 0.704 | 0.681 |

**S2 Table. C-statistics from cross validation by measurement length and temporal resolution - Sensitivity analysis without restricting the wash-in period.**

| **Measurement length [Minutes]** | **Temporal resolution [Seconds]** | | | | | | |
| --- | --- | --- | --- | --- | --- | --- | --- |
|  | **1.6** | **3.2** | **4.8** | **6.4** | **8.0** | **16.0** | **30.4** |
| 2.0 | 0.691 | 0.677 | 0.693 | 0.679 | 0.710 | 0.714 | 0.709 |
| 2.5 | 0.712 | 0.700 | 0.719 | 0.695 | 0.720 | 0.720 | 0.696 |
| 3.0 | 0.671 | 0.683 | 0.663 | 0.689 | 0.686 | 0.665 | 0.662 |
| 3.5 | 0.695 | 0.700 | 0.685 | 0.711 | 0.691 | 0.685 | 0.678 |
| 4.0 | 0.688 | 0.719 | 0.681 | 0.720 | 0.730 | 0.690 | 0.664 |
| 4.5 | 0.694 | 0.707 | 0.717 | 0.726 | 0.731 | 0.706 | 0.661 |
| 5.0 | 0.683 | 0.691 | 0.680 | 0.735 | 0.695 | 0.708 | 0.684 |
| 5.5 | 0.697 | 0.681 | 0.670 | 0.718 | 0.697 | 0.704 | 0.671 |
| 6.0 | 0.690 | 0.694 | 0.672 | 0.700 | 0.685 | 0.686 | 0.697 |
| 6.5 | 0.694 | 0.696 | 0.698 | 0.710 | 0.705 | 0.686 | 0.664 |
| 7.0 | 0.709 | 0.683 | 0.685 | 0.694 | 0.723 | 0.686 | 0.682 |
| 7.5 | 0.700 | 0.702 | 0.686 | 0.714 | 0.701 | 0.709 | 0.692 |
| 8.0 | 0.695 | 0.731 | 0.695 | 0.697 | 0.691 | 0.717 | 0.714 |
| 10.0 | 0.693 | 0.695 | 0.685 | 0.706 | 0.708 | 0.714 | 0.685 |
| 12.0 | 0.686 | 0.693 | 0.680 | 0.704 | 0.693 | 0.690 | 0.677 |
| 13.0 | 0.724 | 0.685 | 0.669 | 0.707 | 0.750 | 0.677 | 0.666 |

**S3 Table. Impact of measurement length and temporal resolution on
C-statistics from cross validation.**

| **Scenario** | **Measurement length Increase in C-statistics per additional minute (95%-CI)** | **Temporal resolution Increase in C-statistics per additional second (95%-CI)** |
| --- | --- | --- |
| Base analysis | -0.000228 (-0.001134 to 0.000678) | 0.000230 (-0.000074 to 0.000535) |
| Sensitivity analyses |  |  |
| Best model | 0.000554 (-0.000757 to 0.001865) | 0.000445 (0.000004 to 0.000886) |
| Without restriction of wash-in period | 0.000044 (-0.000975 to 0.001064) | -0.000482 (-0.000825 to -0.000139) |
