# Supplementary figures and images for: Optimal length and temporal resolution of dynamic contrast-enhanced MR imaging for the differentiation between prostate cancer and normal peripheral zone tissue
Source: PLoS One. 2023 Jun 23;18(6):e0287651. doi: 10.1371/journal.pone.0287651 (PMC10289347; doi:10.1371/journal.pone.0287651)

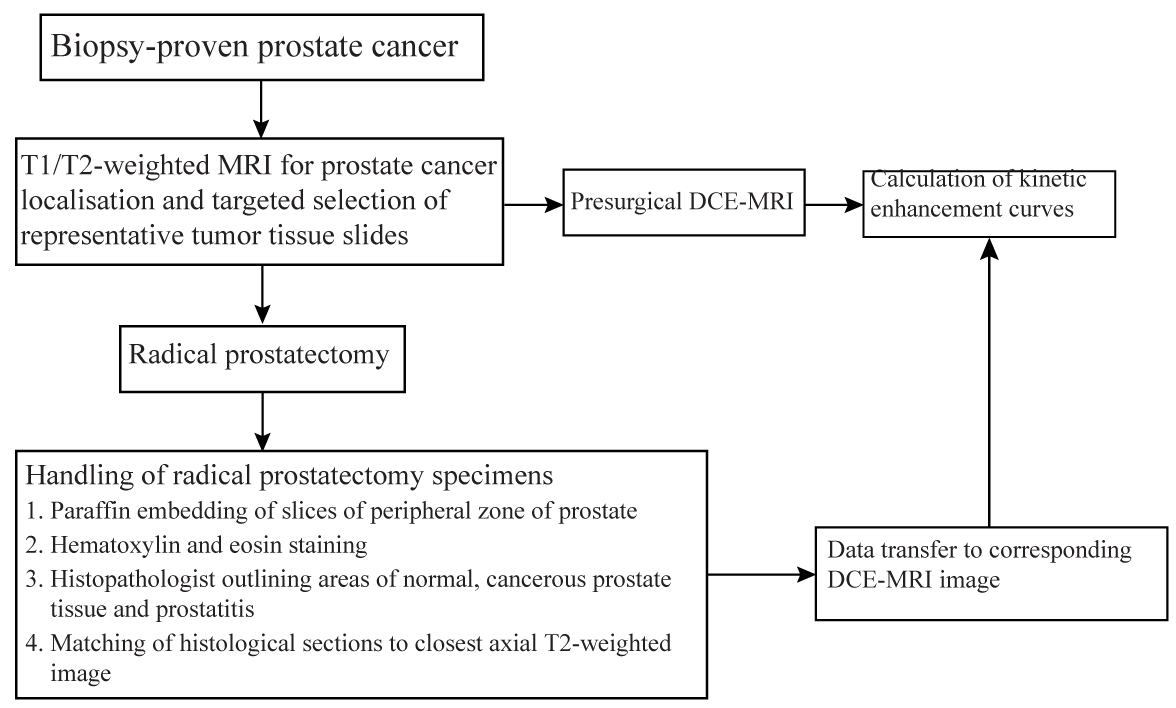

Supplement: S1 Fig — (TIF) [file pone.0287651.s002.tif]
