# Supplementary material for: Optimal length and temporal resolution of dynamic contrast-enhanced MR imaging for the differentiation between prostate cancer and normal peripheral zone tissue
Source: PLoS One. 2023 Jun 23;18(6):e0287651. doi: 10.1371/journal.pone.0287651 (PMC10289347; doi:10.1371/journal.pone.0287651)
Supplement: Attachment — Submitted filename: PLOS ONE review.docx [file pone.0287651.s006.docx]

Thank you very much for selecting me as a reviewer for the article “Optimal length and temporal resolution of dynamic contrast-enhanced MR imaging for the differentiation between prostate cancer and normal peripheral zone tissue”.

This paper deals with very interesting topic of dynamic contrast-enhanced magnetic resonance which is an insufficiently used method and there is a great scientific interest in increasing usability of this method. Problem with the DCE-MRI is lack of standardisation between institutions.

The authors stated that “There are currently insufficient peer reviewed published data or expert consensus to support routine adoption of DCE-MRI for clinical use“ which is only partially correct. DCE-MRI is included in all PI RADS version. According to currently valid PI RADS v2.1 DCE-MRI is used as quantitative method so it is in clinical use. There is insufficient data on semiquantitative and quantitative DCE-MRI, that is , there is a need for research that presents the superiority of these two methods and research that deals with the standardisation of these methods.

Also the authors, through the abstract, introduction and discussion, conclude about the superiority of the non-parametric semiquantitative method compared to the quantitative method, however, as they stated in the limitations of the research, they did not compare the two methods.

There are many research, not only regarding prostate but also many other organ systems, published in high quality journals that support use of quantitative method od DCE-MRI wherever possible. Quantitative study by observing the movement of contrast medium between intra- and extravascular space gives information about tumour angiogenesis so it has a role not only in locoregional staging of the disease or potential role in PI RADS categorisation but also in monitoring of oncologic treatment response.

Methodology is explained in detail, which is the major strength of this manuscript in order to guide further research on this topic. The research is conceptually designed and developed with high quality.

The results are certainly of great value in directing further research.

I hope my comment would be of help for the authors to make their study more suitable for publication.

Thank you.
