# Supplementary material for: Optimal length and temporal resolution of dynamic contrast-enhanced MR imaging for the differentiation between prostate cancer and normal peripheral zone tissue
Source: PLoS One. 2023 Jun 23;18(6):e0287651. doi: 10.1371/journal.pone.0287651 (PMC10289347; doi:10.1371/journal.pone.0287651)
Supplement: Attachment — Submitted filename: Response to Reviewers.docx [file pone.0287651.s007.docx]

Responses to Reviewers

Reviewer comment #1:

Provide the details of the radiological pathological correlation and how they differentiated prostate cancer tissue and normal prostate tissue.

Authors’ response:

Thank you for bringing this to our attention. Details of the radiological pathological correlation and how they differentiated prostate cancer tissue and normal prostate tissue of the study subjects specimens have been published previously. However, our summary of the “Handling of radical prostatectomy specimens” in the methods section has not referenced earlier publications with the details requested.

Following the reviewer’s comment we have expanded the description of the handling of radical prostatectomy specimens in the methods section.

Added text in methods section:

"Details of the histopathologic analysis and its correlation with cancer tissue and normal prostate tissue have been published previously.(1, 2) In summary, an experienced pathologist prepared and assessed all prostatectomy specimens. Slices were perpendicular to the long axis of the gland for optimal correlation with the T2-weighted images and the DCE-MRI slice, which were also axially angulated perpendicular to the long axis and the DCE-MRI slice. Slice orientations and corresponding paraffin blocks were recorded on a chart. Together with a radiologist, the pathologist selected paraffin blocks that matched the DCE-MRI slices. For this they used a pathologic diagram, coronal T2-weighted images and sagittal localizer images. The urethra was used as a landmark for aligning axial T2-weighted images with their corresponding paraffin blocks. The resulting paraffin blocks were selected jointly by the pathologist and radiologist, corresponding to transversal T2-weighted-images.The selected blocks were cut into 4 µm sections and stained with haematoxylin and eosin."

Reviewer comment #2:

Please provide other prostate MRI findings in addition to the DCE-MRI.

Authors’ response:

We appreciate this suggestion. Other findings in T2W images include areas of chronic prostatitis with band-like, wedge-like, or diffuse T2w hypointensity with corresponding inflammatory lymphocyte and plasma cell infiltrates. However, areas of chronic prostatitis were not further investigated as the aim of this study was to differentiate cancerous from normal prostate tissue.

No changes to the manuscript were applied.

Reviewer comment #3:

Please compare the non-parametric semiquantitative method with the quantitative method.

Authors’ response:

Thank you for this comment. As requested by the reviewer, we have conducted a comparison of the non-parametric semiquantitative method with quantitative parameters from each of the 97 prostate tissue specimens. Quantitative parameters in prostate cancer and normal prostate tissues respectively were blood volume, mean transit time, perfusion, and extravascular volume. We applied the same statistical approach as for the non-parametric semiquantitative method to predict prostate cancer diagnosis. We performed a multivariate logistic regression model with stepwise selection of independent variables (the 4 quantitative parameters) and prostate cancer as a dichotomous dependent variable, developed and derived a score to predict prostate cancer as the sum of logits of the remaining independent variables in the final model, and calculated a C-statistics based on leave-one-pair-out cross-validation.

In normal prostate healthy tissue, mean blood volume (0.007 versus 0.010%), extravascular volume (0.240 versus 0.307%) and perfusion (0.357 versus 0.702 mL/min/cm³) were lower than in prostate cancer tissue, whereas mean transit time was higher in normal prostate tissue than in prostate cancer tissue (2.819 versus 2.303 seconds).

After applying the stepwise approach, the score to predict prostate cancer based on quantitative parameters included the variables blood volume (increasing risk with increasing volume) and transit time (increasing risk with decreasing time). The C-statistics of this model was 0.656 compared to 0.743 of the non-parametric semiquantitative model.

The comparison of the non-parametric semiquantitative method with the quantitative method required the following manuscript changes:

Abstract:

[...]

Thus, the objective of this study was to explore the optimal temporal resolution and measurement length for DCE-MRI to differentiate cancerous from normal prostate tissue of the peripheral zone of the prostate by non-parametric MRI analysis and to compare with a quantitative MRI analysis.

[...]

The optimal C-statistics was 0.743 for temporal resolution of 8.0 seconds and measurement length of 2.5 minutes compared with 0.656 derived from a quantitative MRI analysis.

Introduction:

[...]

The aim of this study was to evaluate the optimal temporal resolution and measurement length for DCE-MRI to differentiate cancerous from normal prostate tissue of the peripheral zone of the prostate by non-parametric MRI analysis and to compare with quantitative MRI analysis.

Methods, subheading Postprocessing DCE-MRI Datasets:

[...]

Furthermore, quantitative parameters were derived from parametric maps.(3) For further data collection, the data visualization software Amira® version: 5.3.3 was used. The relevant ROI on the T2-weighted image was drawn and synchronized with parametric maps for mean blood volume, mean transit time, mean perfusion, and mean extravascular volume.

Methods, subheading Terms and measures:

[...]

For the comparison with the quantitative method, the following quantitative parameters were investigated: mean blood volume, mean transit time, mean perfusion, and mean extravascular volume.

Methods, subheading Data analysis:

[...]

For comparison, we calculated mean values of blood volume, transit time, perfusion, and extravascular volume in normal and prostate cancer tissues respectively. We performed a multivariate logistic regression with stepwise selection of independent variables using prostate cancer as a dichotomous dependent variable and defined a score to predict prostate cancer as the sum of logits of the remaining independent variables in the final model, and calculated a C-statistics based on cross validation using the leave one pair out method.

[...]

Results:

[...]

In normal prostate tissue, mean blood volume (0.007 versus 0.010%), mean extravascular volume (0.240% versus 0.307%) and mean perfusion (0.357 versus 0.702 mL/min/cm³) were lower than in prostate cancer tissue, whereas mean transit time was higher in normal tissue than in prostate cancer tissue (2.819 versus 2.303 seconds). After applying the stepwise approach, the score to predict prostate cancer based on quantitative parameters included the variables blood volume (increasing risk with increasing volume) and transit time (increasing risk with decreasing time). The C-statistics of this model was 0.656 compared to 0.743 of the non-parametric semiquantitative model.

Discussion:

[...]

In contrast, the discriminatory power of the quantitative method yielded a C-statistics of 0.656.

[...]

References

1.	Franiel T, Ludemann L, Rudolph B, Lutterbeck E, Hamm B, Beyersdorff D. Differentiation of prostate cancer from normal prostate tissue: role of hotspots in pharmacokinetic MRI and histologic evaluation. AJR Am J Roentgenol. 2010;194(3):675-81.

2.	Franiel T, Ludemann L, Rudolph B, Rehbein H, Stephan C, Taupitz M, et al. Prostate MR imaging: tissue characterization with pharmacokinetic volume and blood flow parameters and correlation with histologic parameters. Radiology. 2009;252(1):101-8.

3.	Ludemann L, Prochnow D, Rohlfing T, Franiel T, Warmuth C, Taupitz M, et al. Simultaneous quantification of perfusion and permeability in the prostate using dynamic contrast-enhanced magnetic resonance imaging with an inversion-prepared dual-contrast sequence. Ann Biomed Eng. 2009;37(4):749-62.
